# Comparison of Surface Roughness of Different Orthodontic Archwires: Atomic Force Microscopic Study

**DOI:** 10.7759/cureus.51516

**Published:** 2024-01-02

**Authors:** Syed Ashique Abdulhameed, Brijesh S, Jose Sunny, Dwijesh S Goswami, Nevin Abraham, Marie Asha Ambroise

**Affiliations:** 1 Department of Orthodontics and Dentofacial Orthopaedics, Meenakshi Ammal Dental College and Hospital, Chennai, IND; 2 Department of Orthodontics and Dentofacial Orthopaedics, Mar Baselios Dental College, Ernakulam, IND; 3 Department of Orthodontics and Dentofacial Orthopaedics, Al Azhar Dental College, Thodupuzha, IND; 4 Department of Orthodontics and Dentofacial Orthopaedics, Ahemdabad Dental College and Hospital, Ahmedabad, IND; 5 Department of Orthodontics and Dentofacial Orthopaedics, Pushpagiri College of Dental Sciences, Thiruvalla, IND; 6 Department of Orthodontics and Dentofacial Orthopaedics, Sri Venkateshwaraa Dental College, Puducherry, IND

**Keywords:** fixed orthodontics, orthodontics, afm, orthodontic archwires, surface roughness

## Abstract

Background: Surface roughness (SR) of dental components is significant because it impacts the surface area of the contact surface, which in turn affects corrosion behavior and biological compatibility. Orthodontic archwires (OA) with SR can affect the coefficient of friction, which in turn affects how effectively sliding biomechanics work and how the orthodontic appliance works efficiently.

Aim: The objectives of the present investigation were to examine the SR of five distinct kinds of OA using an atomic force microscope (AFM) and to assess the merits of using AFM to analyze orthodontic materials.

Methods: For this investigation, there were five distinct orthodontic archwires with rectangular cross-sectional geometry. There were assigned different categories: Category 1: SmartArch wires (Ormco) (n=20), Category 2: Damon wires (Ormco) (n=20), Category 3: heat-activated nickel-titanium (HANT) wires (G&H Orthodontics) (n=20), Category 4: nickel-titanium (NiTi) wires (G&H Orthodontics) (n=20), Category 5: stainless steel (SS) wires (Ormco) (n=20). Each wire category's 20 samples were selected. Ten samples from each category had 5 mm of wire clipped from the finish point of the archwires. These were observed using the AFM in natural lighting. Using a cyanoacrylate glue that dries quickly, the samples were fastened to a metal holder. Ten randomly chosen patches of the surface, each measuring 15 × 15 µm, were taken from every sample and examined (N = 500).

Results: The mean values of roughness average (Ra) in category 1, category 2, category 3, category 4 and category 5 were 23.08 ± 17.66, 26.78 ± 5.65, 26.66 ± 3.89, 9.71 ± 0.29 and 11.29 ± 2.12 respectively. The values of Ra representing SR were greatest in category 3 (HANT wires) followed by category 2 (Damon wires) while values of SR were minimum in category 4 (NiTi wires) and category 5 (stainless steel wires). The findings had statistical significance also.

Conclusion: The SR of stainless steel wire was discovered to be less than that of the other wires. The SR may have an impact on the efficiency of sliding mechanics as well as the appeal and corrosion resistance of orthodontic components.

## Introduction

Dental materials must be sufficiently biocompatible in the adverse setting and able to survive the thermal, mechanical, and chemical pressures in the oral cavity of the patient. Because this affects the area of the interaction surface, which in turn affects corrosion behavior and biological compatibility, the surface value, or surface roughness (SR), of dental components is crucial [1]. By influencing the value of the coefficient of friction, SR of orthodontics archwires can also have an impact on the appearance of the orthodontic appliance and the effectiveness of sliding biomechanics [2,3].

When an orthodontic archwire is used to guide a tooth into its desired position, the tooth may undergo tipping and rotational movements, ultimately coming into contact with both the orthodontic bracket and the guiding orthodontic archwire. As a result, frictional stresses may result in a 50% or greater reduction in force applied through orthodontic appliances [4,5]. The loss caused by friction is influenced by a wide range of mechanical properties of the orthodontic archwires (OA) and orthodontic brackets being utilized. However, the guiding archwire's characteristics of the material are the most significant variables. 

In addition, the SR of the alloy is one of its properties that affects how the archwires behave. OA effectiveness and biological compatibility are both impacted by surface properties, according to studies [6]. Surface texture can also play a significant role in determining the resistance to corrosion, the visual appeal, and the overall success of orthodontic devices [7]. In addition, SR fluctuation has an impact on plaque accumulation, which in turn has a significant impact on the other qualities mentioned before. Primarily everything else, the coefficient of friction can be altered by SR [8,9].

Surface roughness in metals is intrinsic and mostly caused by tiny asperities. The nominal interface area is much larger than the actual contact area between two solid surfaces on a microscopic scale. The precise definition of the effective area is the total area of contact between the minuscule imperfections on the surfaces. Known as asperities, these tiny points take on the task of supporting the whole weight distributed throughout the surfaces [10].

The examination of SR of the various OA available in the market is thus a crucial stage in the assessment of archwire effectiveness. Surface profilometry, during which a thin tip has been employed to look at the contours in just one single line of a defined area, was once the primary method for determining SR [10]. The main disadvantage of this technology was the inability to measure surface flaws close to the scan plane. In addition, profilometry was traumatic, and scratches on the surface while scanning was an issue. Thus, the growing need for not harmful and minimally invasive procedures has improved new analytical approaches built on existing optical approaches and scanning tunneling microscopes [11,12]. These techniques allow for the indirect scanning of a surface region of model that has been chosen. The atomic force microscope (AFM) is included in scanning probe imaging [13]. Because it can offer three-dimensional details regarding surface morphological features the AFM is regarded as the most suitable technique to assess surface topography [14]. 

The objectives of the present investigation were to examine SR of five distinct kinds of OA using AFM and to assess the merits of using AFM to analyze orthodontic materials.

## Materials and methods

Study specimens

For this investigation, a total of 100 orthodontic archwire specimens were used, which were categorized into five distinct groups based on their rectangular cross-sectional geometry. There were assigned different categories as Category 1: SmartArch wires (Ormco, Brea, CA, USA) (n=20), Category 2: Damon wires (Ormco) (n=20), Category 3: Heat-activated nickel-titanium (HANT) wires (G&H Orthodontics, Franklin, IN, USA) (n=20), Category 4: Nickel-titanium (NiTi) wires (G&H Orthodontics) (n=20), Category 5: Stainless steel (SS) wires (Ormco) (n=20).

**Figure 1:**
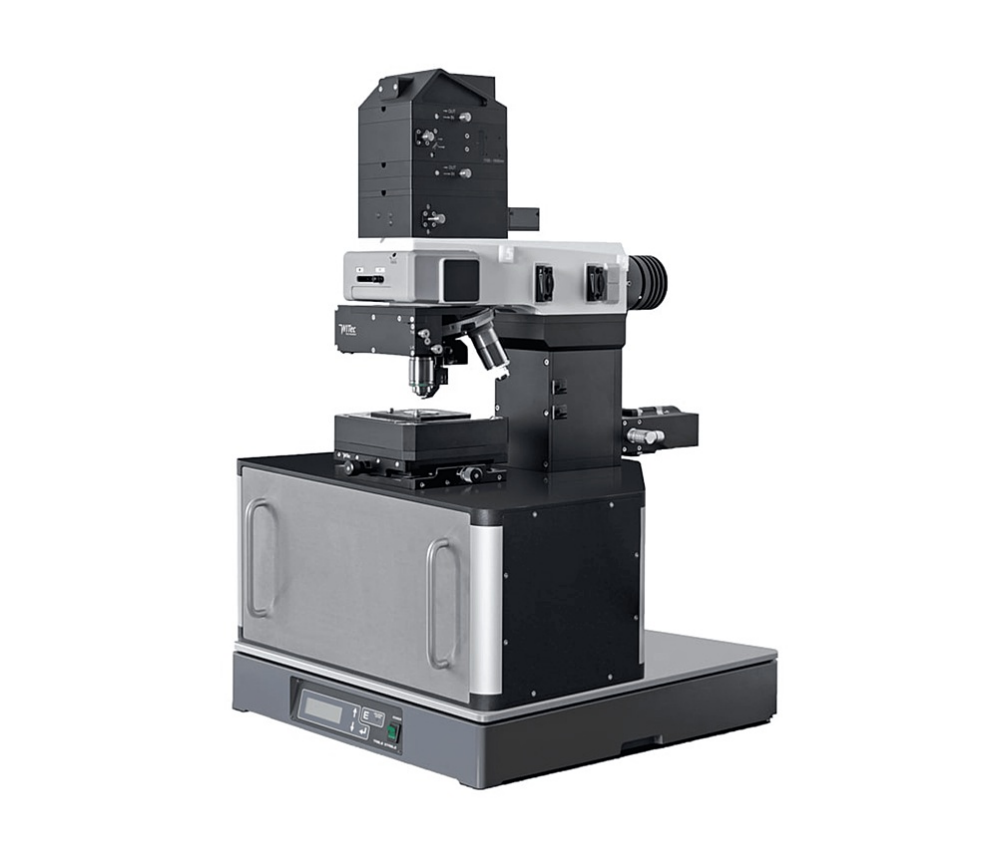
Atomic force microscope used

Each wire category's 20 samples were selected. Ten samples from each category had 5 mm of wire clipped from the finish point of the archwires. These were observed using the AFM in natural lighting. Using a cyanoacrylate glue that dries quickly, the samples were fastened to a metal holder. Ten randomly chosen patches of the surface, each measuring 15 × 15 µm, were taken from every sample and examined (N = 500) (Figure 1).

Settings of AFM

**Figure 2:**
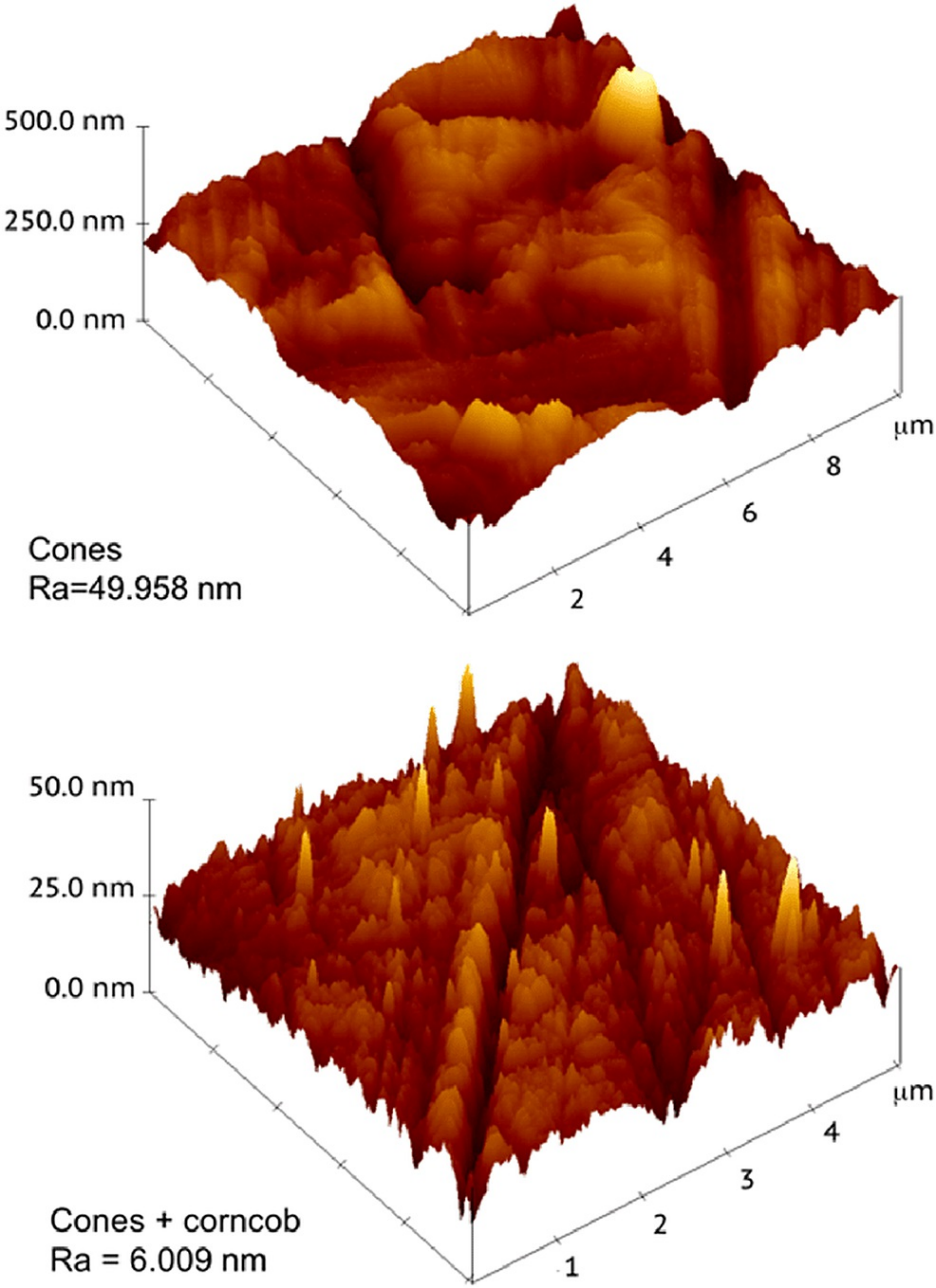
Atomic force microscope results on the software showing the surface characteristics Ra: Average surface roughness of scanned surface

We employed AFM probes with 10 nm curvature radii installed on cantilevers of 250 mm length with 0.1 N/m spring constants. The photographs in three dimensions were processed using the Gwyddion program. The software evaluated the following variables: average surface roughness of the scanned surface (Ra), root mean square of the scanned surface (rms), and the highest point of the surface profile apex denoted by mh. The Ra and rms represent the arithmetical mean of the absolute values and the root mean square value of the scanned surface profile, respectively; mh is the maximum height of a profile peak (Figure 2).

Statistical analysis

For the statistical analysis of the surface features of the various orthodontic archwires, SPSS version 23.0.0 (IBM Corp., Armonk, NY, USA) was employed. The analysis included descriptive statistics to summarize the data, one-way ANOVA to determine if there were significant differences among the wire categories, and post hoc Tukey testing to identify specific pairwise differences between categories. This comprehensive approach allowed for a thorough examination of the surface characteristics of the orthodontic archwires and their potential implications.

## Results

The mean values of Ra in category 1, category 2, category 3, category 4, and category 5 were 23.08 ± 17.66, 26.78 ± 5.65, 26.66 ± 3.89, 9.71 ± 0.29, and 11.29 ± 2.12 respectively. The values of Ra representing SR were greatest in category 3 (HANT wires) followed by category 2 (Damon wires) while values of SR were minimum in category 4 (NiTi wires) and category 5 (stainless steel wires). The findings had statistical significance also.

The mean values of rms in category 1, category 2, category 3, category 4 and category 5 was 21.42 ± 7.49, 35.42 ± 9.83, 33.36 ± 6.15, 15.50 ± 1.72 and 16.11 ± 4.61 respectively. The values of rms were greatest in category 2 (Damon wires) followed by category 3 (HANT wires) while values of rms were minimum in category 4 (NiTi wires) and category 5 (stainless steel wires). The statistical analysis gave significant variations in rms values in different OA.

The mean values of mh in category 1, category 2, category 3, category 4 and category 5 were 287.79 ± 65.68, 259.27 ± 64.40, 367.64 ± 90.52, 209.41 ± 57.42 and 171.16 ± 35.73 respectively. The values of mh were greatest in category 3 (HANT wires) followed by category 1 (SmartArch wires) while values of mh were minimum in category 4 (NiTi wires) and category 5 (stainless steel wires). The statistical analysis gave significant variations in rms values in different OA (Table 1).

**Table 1:** Values of Ra, rms and mh in five different categories of OA Ra: Average surface roughness of scanned surface; rms: Root mean square of scanned surface; mh: Highest point of the surface profile apex; OA: Orthodontic archwires; SD: Standard deviation. Category 1: SmartArch wires (Ormco), Category 2: Damon wires (Ormco), Category 3: Heat-activated nickel-titanium (HANT) wires (G&H Orthodontics), Category 4: Nickel-titanium (NiTi) wires (G&H Orthodontics), Category 5: Stainless steel (SS) wires (Ormco).

Variable	Category 1	Category 2	Category 3	Category 4	Category 5
Ra, Mean±SD	23.08 ± 17.66	26.78 ± 5.65	26.66 ± 3.89	9.71 ± 0.29	11.29 ± 2.12
rms, Mean±S.D	21.42 ± 7.49	35.42 ± 9.83	33.36 ± 6.15	15.50 ± 1.72	16.11 ± 4.61
mh, Mean±SD	287.79 ± 65.68	259.27 ± 64.40	367.64 ± 90.52	209.41 ± 57.42	171.16 ± 35.73

Table 2 displays statistical comparisons among various categories (category 1, category 2, category 3, category 4, and category 5) across three distinct variables (Ra, rms, mh). 

**Table 2:** Archwire roughness parameters statistical analysis P values Ra: Average surface roughness of scanned surface; rms: Root mean square of scanned surface; mh: Highest point of the surface profile apex. Category 1: SmartArch wires (Ormco), Category 2: Damon wires (Ormco), Category 3: Heat-activated nickel-titanium (HANT) wires (G&H Orthodontics), Category 4: Nickel-titanium (NiTi) wires (G&H Orthodontics), Category 5: Stainless steel (SS) wires (Ormco).

Variable	Ra	rms	mh
Category 1 vs Category 2	P> 0.005	P<0.001	P>0.005
Category 1 vs Category 3	P>0.005	P<0.001	P>0.005
Category 1 vs Category 4	P<0.01	p>0.005	P<0.05
Category 1 vs Category 5	P<0.01	p>0.005	p>0.005
Category 2 vs category 3	P>0.005	p>0.005	P<0.01
Category 2 vs category 4	P<0.001	P<0.001	P<0.05
Category 2 vs category 5	P<0.001	P<0.001	p>0.005
Category 3 vs category 4	P<0.001	P<0.001	P<0.001
Category 3 vs category 5	P<0.001	P<0.001	P<0.001
Category 4 vs category 5	P>0.005	P>0.005	P>0.005

When comparing category 1 to category 2 and category 3, the variables rms showed statistically significant differences with p-values less than 0.001, signifying substantial variations. Comparing category 1 to category 4 revealed statistical significance for Ra (p < 0.01) and mh (p < 0.05), the category 1 versus category 5 comparison demonstrated statistical significance for Ra (p < 0.01). Furthermore, category 2 versus category 4 showed high significance across all three variables. Category 2 versus category 5 showed high statistical significance for Ra and rms (both p < 0.001). Overall, the p-values provide valuable insights into the statistical relationships between the categories for each variable. Comparing category 3 to category 4 and category 3 to category 5 yielded high statistical significance for all three variables (p < 0.001).

These findings highlight the unique relationships and disparities between the categories with regard to Ra, rms, and mh. The variations in statistical significance levels help elucidate which differences are substantial and which are not, providing valuable insights into the dataset's characteristics and potential implications for the study's objectives.

## Discussion

The examination of SR of the various OA available in the market is a crucial stage in the assessment of archwire effectiveness [15-18]. The objectives of the present investigation were to examine the SR of five distinct kinds of OA using atomic force micros and to assess the merits of using AFM to analyze orthodontic materials. In this study the mean values of Ra in category 1, category 2, category 3, category 4 and category 5 were 23.08 ± 17.66, 26.78 ± 5.65, 26.66 ± 3.89, 9.71 ± 0.29 and 11.29 ± 2.12 respectively. The values of Ra representing SR was greatest in category 3 (HANT wires) followed by category 2 (Damon wires) while values of SR were minimum in category 4 (NiTi wires) and category 5 (stainless steel wires). The findings had statistical significance also.

The SR of titanium molybdenum alloy (TMA) coated wires, TMA and NiTi and NiTi-coated wires and other materials have been compared in various investigations [15]. The surface characteristics of NiTi orthodontic wires, shape memory orthodontic, stainless steel have been compared in fewer investigations [16-18]. In this investigation, stainless steel showed minimum SR. AFM technique was used by D'Ant et al. [15] to make comparisons among one stainless steel orthodontic wire, three beta titanium orthodontic wires and four NiTi orthodontic wires. Copper NiTi wires, nitinol (NiTi), titanium molybdenum alloy (TMA) and stainless steel alloy orthodontic wires were contrasted by Yousif and Abd El-Karim [19] in their study. According to findings from both trials, stainless steel OA exhibited the lowest SR, frictional coefficient and sliding resistance. 

A dissipative pressure called friction opposes the relative movement of two objects coming into contact [20,21]. It prevents the OB from moving correctly along OA in orthodontic treatment. Molecular bonding, or the electromagnetic interactions between atoms, surface roughness-induced interconnection, and the plowing action are the three main contributors to friction [22,23]. It is noteworthy to emphasize that the friction coefficient (m), as established by the second principle of friction, operates independently of the visible contact area if the surface in question may be reshaped plastically [24,25]. However, a fundamental tenet of the hypothesis of force of friction is that surfaces which appear to be uniformly smooth and flat do not appear smooth when examined at the microscopic level. In reality, metals have a rough surface [26,27]. In this study the mean values of rms in category 1, category 2, category 3, category 4 and category 5 were 21.42 ± 7.49, 35.42 ± 9.83, 33.36 ± 6.15, 15.50 ± 1.72 and 16.11 ± 4.61 respectively. The values of rms were greatest in category 2 (Damon wires) followed by category 2 (HANT wires) while values of rms were minimum in category 4 (NiTi wires) and category 5 (stainless steel wires). The statistical analysis gave significant variations in rms values in different OA.

One of the significant advancements in orthodontic research has been the wide availability and utilization of various alloys in the fabrication of OAs. This progress in material selection has greatly improved mechanotherapy in orthodontics, offering more options and enhancing the effectiveness of orthodontic treatment. The continuous introduction of new materials to orthodontists can occasionally make it more challenging to comprehend the genuine characteristics of the wires. In reality, the widespread claims of improved performance are not always supported by credible statistics. Therefore, it can be claimed that evaluating archwire alloys is the first step in understanding how OA behaves in a clinical environment [15].

There are many qualities that should be considered when searching for the ideal OA, including aesthetics, springback, resilience, weldability, formability, and friction. The primary method for calculating SR was surface profilometry, which uses a small tip to examine the contours in just one single line of a specific area. The inability to measure surface imperfections near to the scan plane was this technology's biggest drawback. Additionally, profilometry proved painful, and surface scratches during scanning were a problem. Thus, new analytical methods improved on existing optical methods 15 and scanning tunneling microscopes have been established in response to the increased demand for non-harmful and minimally invasive operations [19]. These techniques allow for the indirect scanning of a surface region of model that has been chosen. The AFM is included in scanning probe imaging. Because it can offer three-dimensional details regarding surface morphological features the AFM is regarded as the most suitable technique to assess surface topography [15,19]. The mean values of mh in category 1, category 2, category 3, category 4 and category 5 were 287.79 ± 65.68, 259.27 ± 64.40, 367.64 ± 90.52, 209.41 ± 57.42 and 171.16 ± 35.73 respectively. The values of mh were greatest in category 3 (HANT wires) followed by category 1 (SmartArch wires) while values of mh were minimum in category 4 (NiTi wires) and category 5 (stainless steel wires). The statistical analysis gave significant variations in rms values in different OA.

Prososki et al. [20] compared one beta-titanium OA and one SS wire OA with nine NiTi OA while Bourauel et al. [10] compared one beta-titanium OA and one SS wire OA with 11 NiTi OA. The stainless steel OA exhibited the least SR, according to the authors of both trials. Furthermore, Shin et al.'s [21] comparison of stainless steel OA and NiTi OA found that the latter had the least SR. Similar results were also seen by Amini et al. [22], although they were not statistically noteworthy. However, they also claimed that American Orthodontics' NiTi OA had less SR than the stainless steel OA, which was possibly the result of techniques of random sampling. Additionally, the chosen components might have been harmed during production or delivery.

Dental materials must be strong enough to withstand the temperature, mechanical, and chemical stresses present in the patient's oral cavity and be adequately biocompatible in a challenging environment [15]. Surface value, or surface roughness, of dental components, is significant because it impacts the area of the interaction surface, which in turn affects corrosion behavior and biological compatibility [17,18]. Orthodontic archwires with SR can affect the coefficient of friction, which in turn affects how effectively sliding biomechanics work and how the orthodontic appliance looks [19,20]. When a tooth is guided by OA, it undergo tipping movement and rotation movement, coming into contact with the guiding orthodontic bracket (OB) and OA. As a result, the force exerted by an orthodontic appliance may be reduced by at least 50% due to frictional tensions [21,22]. Numerous mechanical characteristics of the used orthodontic brackets and OA have an impact on the loss due to friction. The most important factors, however, are the material properties of the guiding archwire [23,24]. AFM was used in the current investigation to examine the surface topographic properties of archwires. AFM is a well-established, non-invasive alternative to profilometry surface analysis, thus we decided to employ it in our research. There was not a statistically significant distinction between the data obtained from digital optical microscopy approaches and the AFM approach according to research by Yousif and Abd El-Karim [19] comparing the SR of OA acquired using digital optical microscopy and AFM. This suggests that both approaches are useful to estimate the SR of OA.

OA frictional coefficient is influenced by their physical properties and SR [15,16]. An undesirable element that can affect the tooth motion during sliding biomechanics is the force of friction between the OB and OA. There is a link between SR with friction, according to several studies [24,25]. However, the motion of teeth during orthodontic treatment is a complex process involving a number of significant variables. One of the key elements determining the surface topographical features of OA is the production method [26]. It has been noted that the supplied wires' SR for a collection of wires made by the very same manufacturer is nearly identical [27].

The study has some limitations such as study examined the SR of the archwires, but the results may have been influenced by the preparation and handling of the wire samples. Variations in sample preparation could introduce potential sources of error. The study primarily focused on the assessment of SR, which provides insights into the surface characteristics of the archwires. However, the long-term implications of SR on clinical outcomes, such as the actual performance of orthodontic appliances in patient treatment, were not explored. Future research could consider studying the clinical relevance of SR in orthodontics. The study did not explore the potential influence of external factors, such as environmental conditions or patient-specific variables, on the SR of orthodontic archwires. Considering these factors in future research could provide a more holistic perspective on the behavior of these materials in clinical settings.

## Conclusions

The SR of stainless steel wire was discovered to be less than that of the other wires. The SR may have an impact on the efficiency of sliding mechanics as well as the appeal and corrosion resistance of orthodontic components. Therefore, during the development process, the surface smoothness of OA should be improved.
